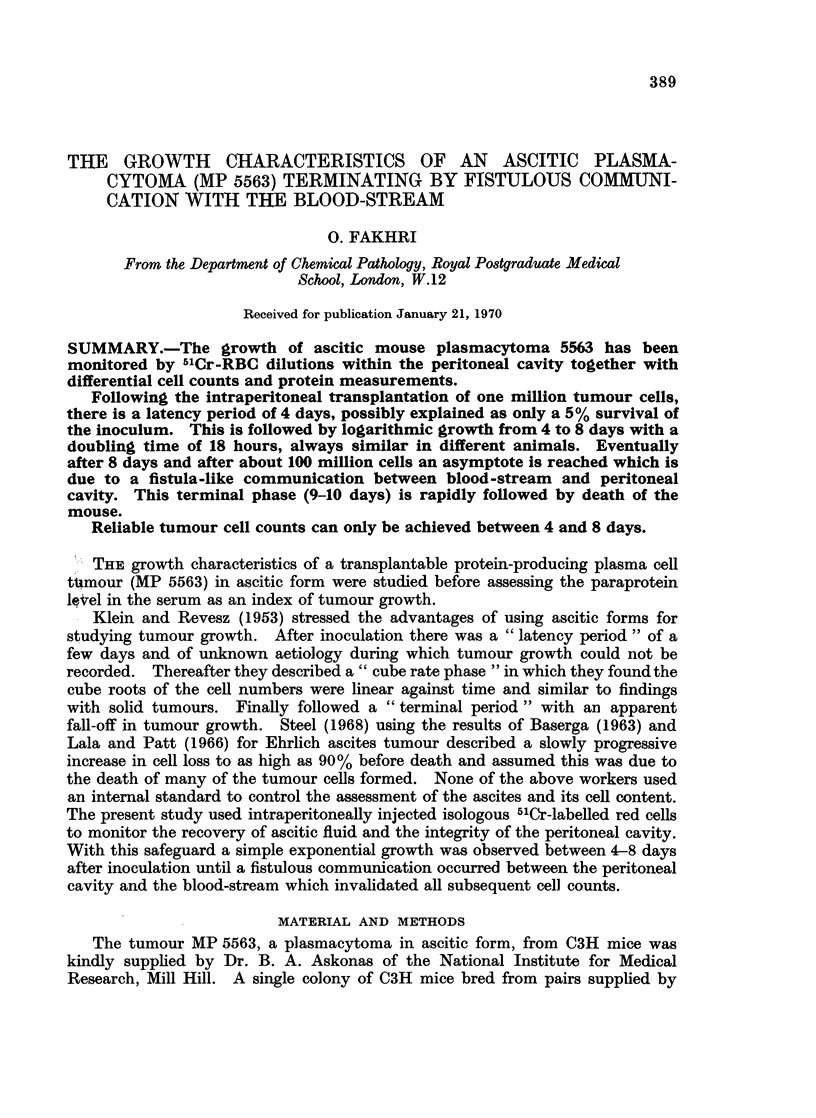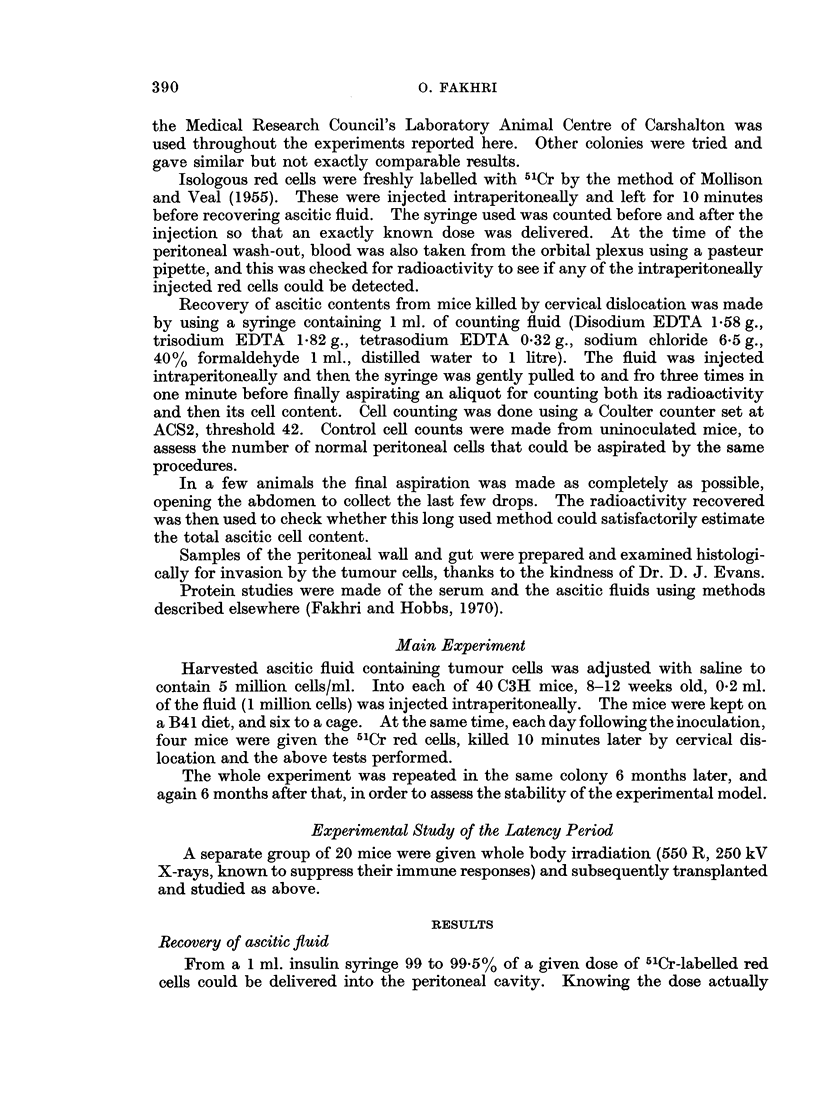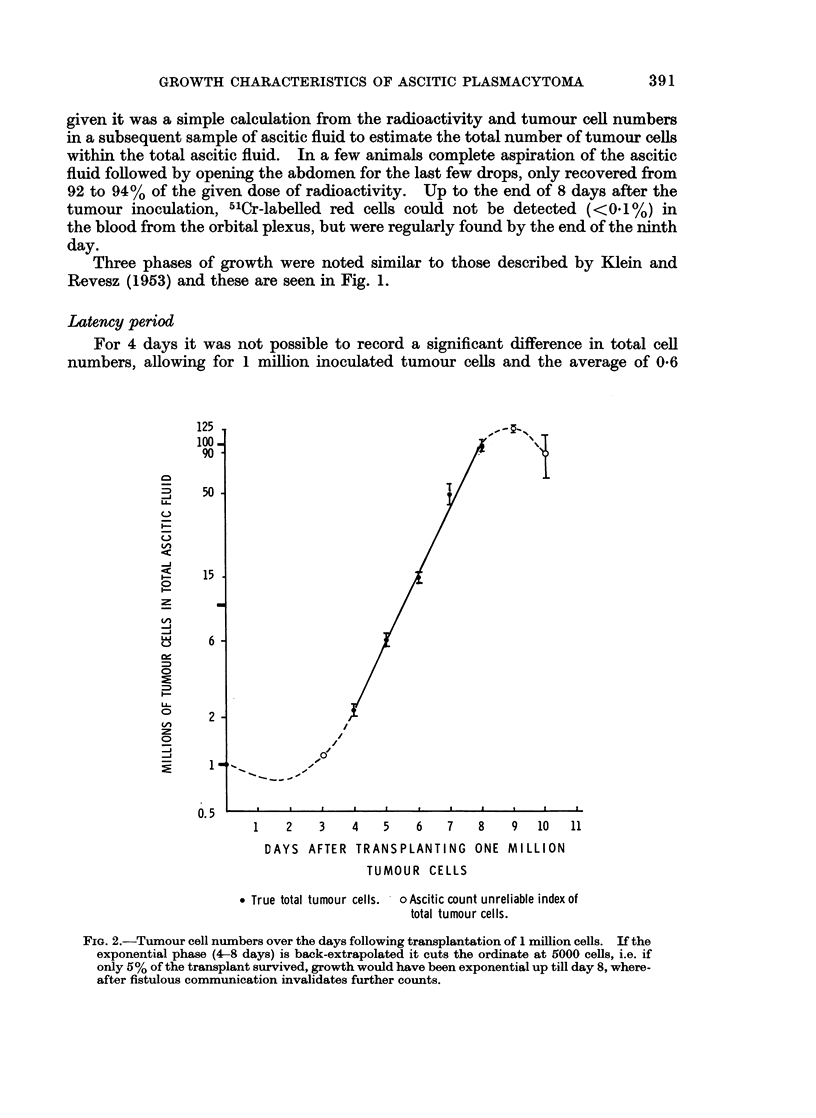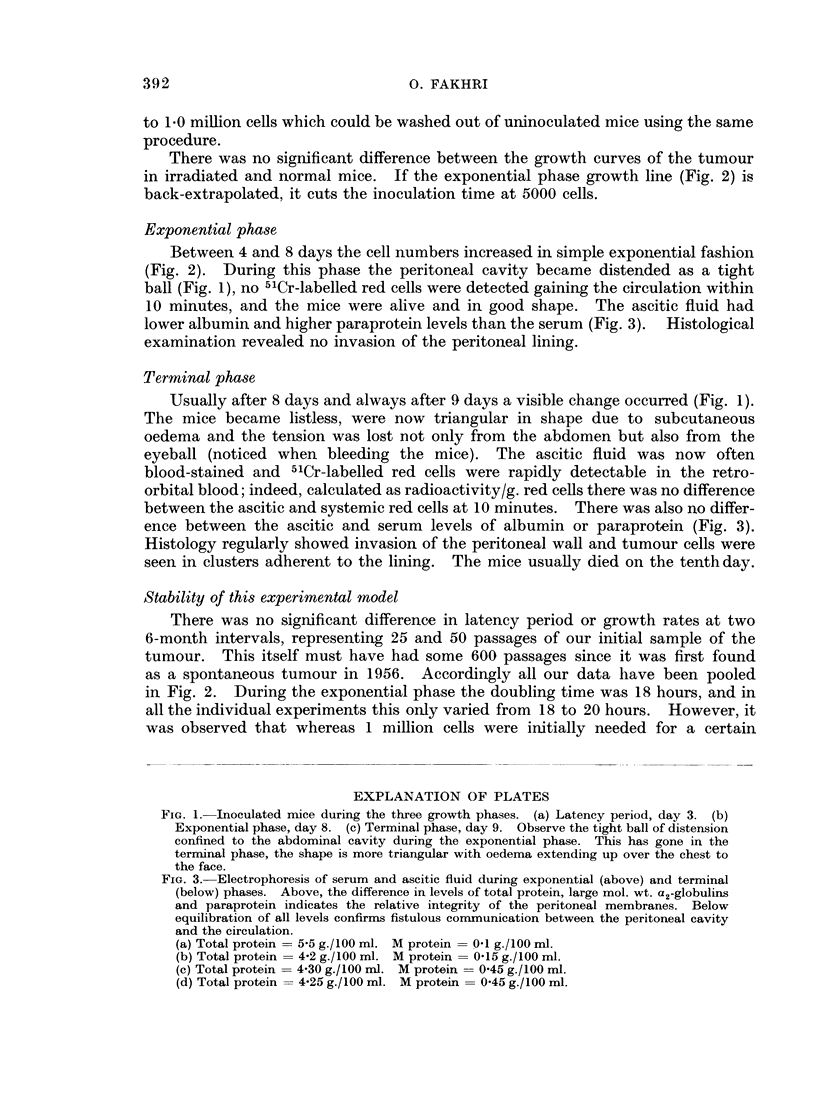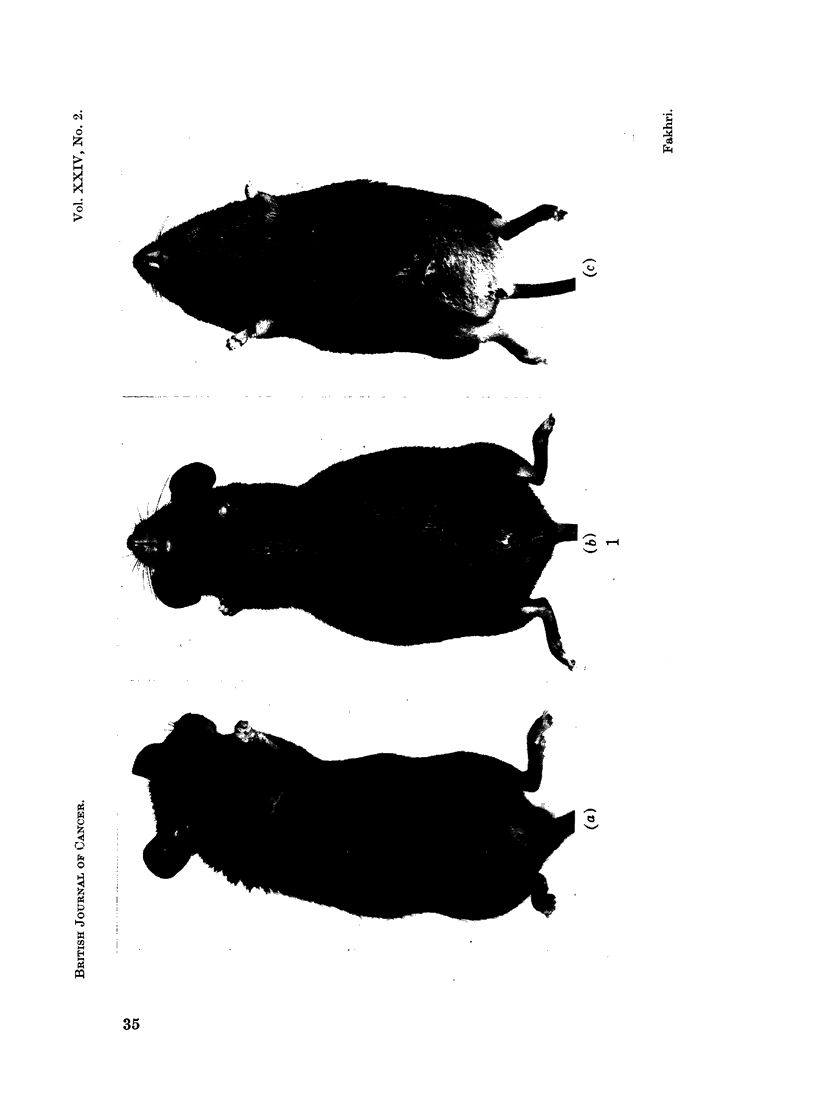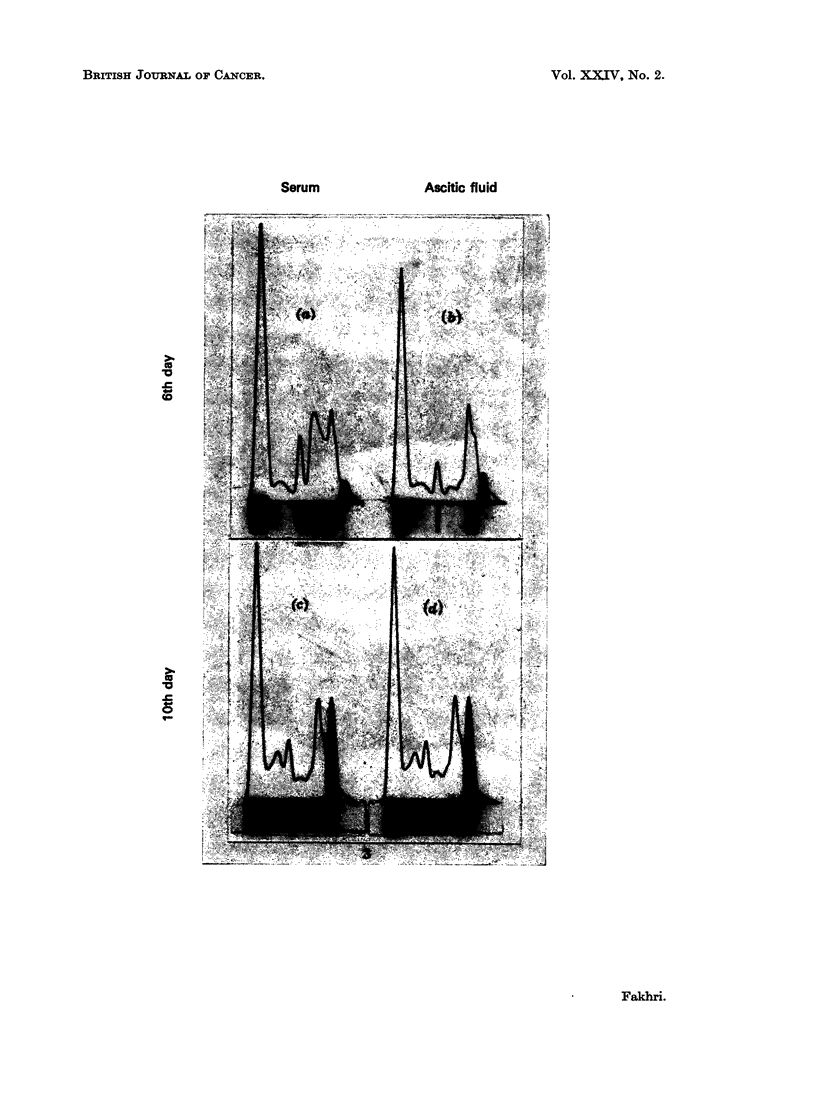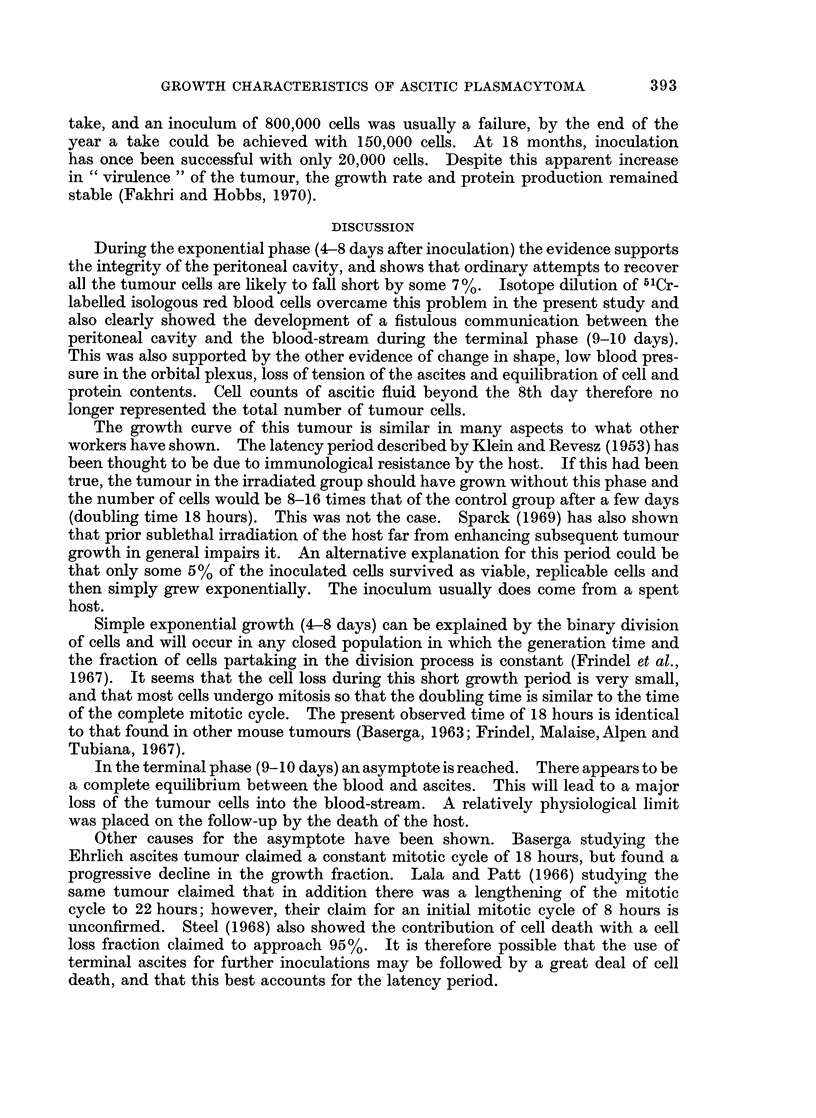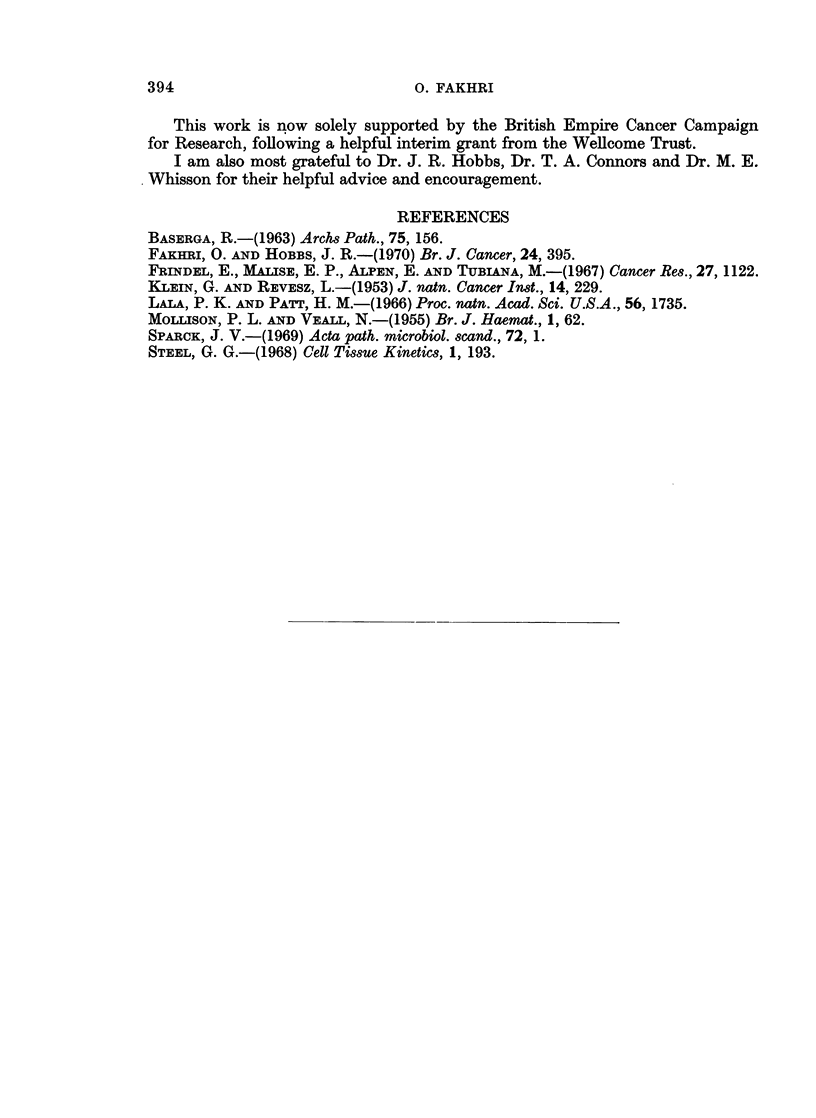# The Growth Characteristics of an Ascitic Plasmacytoma (MP 5563) Terminating by Fistulous Communication with the Blood-Stream

**DOI:** 10.1038/bjc.1970.45

**Published:** 1970-06

**Authors:** O. Fakhri

## Abstract

**Images:**


### THE GROWTH CHARACTERISTICS OF AN ASCITIC PLASMACYTOMA (MP 5563) TERMINATING BY FISTULOUS COMMUNICATION WITH THE BLOOD-STREAM

O. FAKHRI

*From the Department of Chemical Pathology, Royal Postgraduate Medical School, London, W.12*

Received for publication January 21, 1970

**SUMMARY.—The growth of ascitic mouse plasmacytoma 5563 has been monitored by $^{51}$Cr-RBC dilutions within the peritoneal cavity together with differential cell counts and protein measurements.**

**Following the intraperitoneal transplantation of one million tumour cells, there is a latency period of 4 days, possibly explained as only a 5% survival of the inoculum. This is followed by logarithmic growth from 4 to 8 days with a doubling time of 18 hours, always similar in different animals. Eventually after 8 days and after about 100 million cells an asymptote is reached which is due to a fistula-like communication between blood-stream and peritoneal cavity. This terminal phase (9–10 days) is rapidly followed by death of the mouse.**

**Reliable tumour cell counts can only be achieved between 4 and 8 days.**

THE growth characteristics of a transplantable protein-producing plasma cell tumour (MP 5563) in ascitic form were studied before assessing the paraprotein level in the serum as an index of tumour growth.

Klein and Revesz (1953) stressed the advantages of using ascitic forms for studying tumour growth. After inoculation there was a " latency period " of a few days and of unknown aetiology during which tumour growth could not be recorded. Thereafter they described a " cube rate phase " in which they found the cube roots of the cell numbers were linear against time and similar to findings with solid tumours. Finally followed a " terminal period " with an apparent fall-off in tumour growth. Steel (1968) using the results of Baserga (1963) and Lala and Patt (1966) for Ehrlich ascites tumour described a slowly progressive increase in cell loss to as high as 90% before death and assumed this was due to the death of many of the tumour cells formed. None of the above workers used an internal standard to control the assessment of the ascites and its cell content. The present study used intraperitoneally injected isologous $^{51}$Cr-labelled red cells to monitor the recovery of ascitic fluid and the integrity of the peritoneal cavity. With this safeguard a simple exponential growth was observed between 4–8 days after inoculation until a fistulous communication occurred between the peritoneal cavity and the blood-stream which invalidated all subsequent cell counts.

### MATERIAL AND METHODS

The tumour MP 5563, a plasmacytoma in ascitic form, from C3H mice was kindly supplied by Dr. B. A. Askonas of the National Institute for Medical Research, Mill Hill. A single colony of C3H mice bred from pairs supplied by the Medical Research Council's Laboratory Animal Centre of Carshalton was used throughout the experiments reported here. Other colonies were tried and gave similar but not exactly comparable results.

Isologous red cells were freshly labelled with $^{51}Cr$ by the method of Mollison and Veal (1955). These were injected intraperitoneally and left for 10 minutes before recovering ascitic fluid. The syringe used was counted before and after the injection so that an exactly known dose was delivered. At the time of the peritoneal wash-out, blood was also taken from the orbital plexus using a pasteur pipette, and this was checked for radioactivity to see if any of the intraperitoneally injected red cells could be detected.

Recovery of ascitic contents from mice killed by cervical dislocation was made by using a syringe containing 1 ml. of counting fluid (Disodium EDTA 1·58 g., trisodium EDTA 1·82 g., tetrasodium EDTA 0·32 g., sodium chloride 6·5 g., 40% formaldehyde 1 ml., distilled water to 1 litre). The fluid was injected intraperitoneally and then the syringe was gently pulled to and fro three times in one minute before finally aspirating an aliquot for counting both its radioactivity and then its cell content. Cell counting was done using a Coulter counter set at ACS2, threshold 42. Control cell counts were made from uninoculated mice, to assess the number of normal peritoneal cells that could be aspirated by the same procedures.

In a few animals the final aspiration was made as completely as possible, opening the abdomen to collect the last few drops. The radioactivity recovered was then used to check whether this long used method could satisfactorily estimate the total ascitic cell content.

Samples of the peritoneal wall and gut were prepared and examined histologically for invasion by the tumour cells, thanks to the kindness of Dr. D. J. Evans.

Protein studies were made of the serum and the ascitic fluids using methods described elsewhere (Fakhri and Hobbs, 1970).

### *Main Experiment*

Harvested ascitic fluid containing tumour cells was adjusted with saline to contain 5 million cells/ml. Into each of 40 C3H mice, 8–12 weeks old, 0·2 ml. of the fluid (1 million cells) was injected intraperitoneally. The mice were kept on a B41 diet, and six to a cage. At the same time, each day following the inoculation, four mice were given the $^{51}Cr$ red cells, killed 10 minutes later by cervical dislocation and the above tests performed.

The whole experiment was repeated in the same colony 6 months later, and again 6 months after that, in order to assess the stability of the experimental model.

### *Experimental Study of the Latency Period*

A separate group of 20 mice were given whole body irradiation (550 R, 250 kV X-rays, known to suppress their immune responses) and subsequently transplanted and studied as above.

### RESULTS

### *Recovery of ascitic fluid*

From a 1 ml. insulin syringe 99 to 99·5% of a given dose of $^{51}Cr$-labelled red cells could be delivered into the peritoneal cavity. Knowing the dose actually given it was a simple calculation from the radioactivity and tumour cell numbers in a subsequent sample of ascitic fluid to estimate the total number of tumour cells within the total ascitic fluid. In a few animals complete aspiration of the ascitic fluid followed by opening the abdomen for the last few drops, only recovered from 92 to 94% of the given dose of radioactivity. Up to the end of 8 days after the tumour inoculation, $^{51}$Cr-labelled red cells could not be detected (<0·1%) in the blood from the orbital plexus, but were regularly found by the end of the ninth day.

Three phases of growth were noted similar to those described by Klein and Revesz (1953) and these are seen in Fig. 1.

*Latency period*

For 4 days it was not possible to record a significant difference in total cell numbers, allowing for 1 million inoculated tumour cells and the average of 0·6

FIG. 2.—Tumour cell numbers over the days following transplantation of 1 million cells. If the exponential phase (4–8 days) is back-extrapolated it cuts the ordinate at 5000 cells, i.e. if only 5% of the transplant survived, growth would have been exponential up till day 8, whereafter fistulous communication invalidates further counts.

to 1·0 million cells which could be washed out of uninoculated mice using the same procedure.

There was no significant difference between the growth curves of the tumour in irradiated and normal mice. If the exponential phase growth line (Fig. 2) is back-extrapolated, it cuts the inoculation time at 5000 cells.

*Exponential phase*

Between 4 and 8 days the cell numbers increased in simple exponential fashion (Fig. 2). During this phase the peritoneal cavity became distended as a tight ball (Fig. 1), no $^{51}$Cr-labelled red cells were detected gaining the circulation within 10 minutes, and the mice were alive and in good shape. The ascitic fluid had lower albumin and higher paraprotein levels than the serum (Fig. 3). Histological examination revealed no invasion of the peritoneal lining.

*Terminal phase*

Usually after 8 days and always after 9 days a visible change occurred (Fig. 1). The mice became listless, were now triangular in shape due to subcutaneous oedema and the tension was lost not only from the abdomen but also from the eyeball (noticed when bleeding the mice). The ascitic fluid was now often blood-stained and $^{51}$Cr-labelled red cells were rapidly detectable in the retro-orbital blood; indeed, calculated as radioactivity/g. red cells there was no difference between the ascitic and systemic red cells at 10 minutes. There was also no difference between the ascitic and serum levels of albumin or paraprotein (Fig. 3). Histology regularly showed invasion of the peritoneal wall and tumour cells were seen in clusters adherent to the lining. The mice usually died on the tenth day.

*Stability of this experimental model*

There was no significant difference in latency period or growth rates at two 6-month intervals, representing 25 and 50 passages of our initial sample of the tumour. This itself must have had some 600 passages since it was first found as a spontaneous tumour in 1956. Accordingly all our data have been pooled in Fig. 2. During the exponential phase the doubling time was 18 hours, and in all the individual experiments this only varied from 18 to 20 hours. However, it was observed that whereas 1 million cells were initially needed for a certain

---

EXPLANATION OF PLATES

FIG. 1.—Inoculated mice during the three growth phases. (a) Latency period, day 3. (b) Exponential phase, day 8. (c) Terminal phase, day 9. Observe the tight ball of distension confined to the abdominal cavity during the exponential phase. This has gone in the terminal phase, the shape is more triangular with oedema extending up over the chest to the face.

FIG. 3.—Electrophoresis of serum and ascitic fluid during exponential (above) and terminal (below) phases. Above, the difference in levels of total protein, large mol. wt. $\alpha_2$-globulins and paraprotein indicates the relative integrity of the peritoneal membranes. Below equilibration of all levels confirms fistulous communication between the peritoneal cavity and the circulation.

(a) Total protein = 5·5 g./100 ml. M protein = 0·1 g./100 ml.
(b) Total protein = 4·2 g./100 ml. M protein = 0·15 g./100 ml.
(c) Total protein = 4·30 g./100 ml. M protein = 0·45 g./100 ml.
(d) Total protein = 4·25 g./100 ml. M protein = 0·45 g./100 ml.

(a) (b) (c)

1

Fakhri.

3

Fakhri.

take, and an inoculum of 800,000 cells was usually a failure, by the end of the year a take could be achieved with 150,000 cells. At 18 months, inoculation has once been successful with only 20,000 cells. Despite this apparent increase in " virulence " of the tumour, the growth rate and protein production remained stable (Fakhri and Hobbs, 1970).

### DISCUSSION

During the exponential phase (4–8 days after inoculation) the evidence supports the integrity of the peritoneal cavity, and shows that ordinary attempts to recover all the tumour cells are likely to fall short by some 7%. Isotope dilution of $^{51}Cr$-labelled isologous red blood cells overcame this problem in the present study and also clearly showed the development of a fistulous communication between the peritoneal cavity and the blood-stream during the terminal phase (9–10 days). This was also supported by the other evidence of change in shape, low blood pressure in the orbital plexus, loss of tension of the ascites and equilibration of cell and protein contents. Cell counts of ascitic fluid beyond the 8th day therefore no longer represented the total number of tumour cells.

The growth curve of this tumour is similar in many aspects to what other workers have shown. The latency period described by Klein and Revesz (1953) has been thought to be due to immunological resistance by the host. If this had been true, the tumour in the irradiated group should have grown without this phase and the number of cells would be 8–16 times that of the control group after a few days (doubling time 18 hours). This was not the case. Sparck (1969) has also shown that prior sublethal irradiation of the host far from enhancing subsequent tumour growth in general impairs it. An alternative explanation for this period could be that only some 5% of the inoculated cells survived as viable, replicable cells and then simply grew exponentially. The inoculum usually does come from a spent host.

Simple exponential growth (4–8 days) can be explained by the binary division of cells and will occur in any closed population in which the generation time and the fraction of cells partaking in the division process is constant (Frindel *et al.*, 1967). It seems that the cell loss during this short growth period is very small, and that most cells undergo mitosis so that the doubling time is similar to the time of the complete mitotic cycle. The present observed time of 18 hours is identical to that found in other mouse tumours (Baserga, 1963; Frindel, Malaise, Alpen and Tubiana, 1967).

In the terminal phase (9–10 days) an asymptote is reached. There appears to be a complete equilibrium between the blood and ascites. This will lead to a major loss of the tumour cells into the blood-stream. A relatively physiological limit was placed on the follow-up by the death of the host.

Other causes for the asymptote have been shown. Baserga studying the Ehrlich ascites tumour claimed a constant mitotic cycle of 18 hours, but found a progressive decline in the growth fraction. Lala and Patt (1966) studying the same tumour claimed that in addition there was a lengthening of the mitotic cycle to 22 hours; however, their claim for an initial mitotic cycle of 8 hours is unconfirmed. Steel (1968) also showed the contribution of cell death with a cell loss fraction claimed to approach 95%. It is therefore possible that the use of terminal ascites for further inoculations may be followed by a great deal of cell death, and that this best accounts for the latency period.

This work is now solely supported by the British Empire Cancer Campaign for Research, following a helpful interim grant from the Wellcome Trust.

I am also most grateful to Dr. J. R. Hobbs, Dr. T. A. Connors and Dr. M. E. Whisson for their helpful advice and encouragement.

### REFERENCES

Baserga, R.—(1963) *Archs Path.*, **75**, 156.
Fakhri, O. and Hobbs, J. R.—(1970) *Br. J. Cancer*, **24**, 395.
Frindel, E., Malise, E. P., Alpen, E. and Tubiana, M.—(1967) *Cancer Res.*, **27**, 1122.
Klein, G. and Revesz, L.—(1953) *J. natn. Cancer Inst.*, **14**, 229.
Lala, P. K. and Patt, H. M.—(1966) *Proc. natn. Acad. Sci. U.S.A.*, **56**, 1735.
Mollison, P. L. and Veall, N.—(1955) *Br. J. Haemat.*, **1**, 62.
Sparck, J. V.—(1969) *Acta path. microbiol. scand.*, **72**, 1.
Steel, G. G.—(1968) *Cell Tissue Kinetics*, **1**, 193.